# Perceived Gender Ratings for High and Low Scorers on the Autism-Spectrum Quotient Consistent with the Extreme Male Brain Account of Autism

**DOI:** 10.1371/journal.pone.0131780

**Published:** 2015-07-17

**Authors:** Diana Weiting Tan, Suzanna N. Russell-Smith, Jessica M. Simons, Murray T. Maybery, Doris Leung, Honey L. H. Ng, Andrew J. O. Whitehouse

**Affiliations:** 1 Neurocognitive Development Unit, School of Psychology, University of Western Australia, Perth, Western Australia, Australia; 2 Telethon Kids Institute, University of Western Australia, Perth, Western Australia, Australia; Brock University, CANADA

## Abstract

The Extreme Male Brain (EMB) theory posits that autistic traits are linked to excessive exposure to testosterone in utero. While findings from a number of studies are consistent with this theory, other studies have produced contradictory results. For example, some findings suggest that rather than being linked to hypermasculinization for males, or defeminization for females, elevated levels of autistic traits are instead linked to more androgynous physical features. The current study provided further evidence relevant to the EMB and androgony positions by comparing groups of males selected for high or low scores on the Autism-spectrum Quotient (AQ) as to the rated masculinity of their faces and voices, and comparable groups of females as to the rated femininity of their faces and voices. The voices of High-AQ males were rated as more masculine than those of Low-AQ males, while the faces of High-AQ females were rated as less feminine than those of Low-AQ females. There was no effect of AQ group on femininity ratings for female voices or on masculinity ratings for male faces. The results thus provide partial support for a link between high levels of autistic-like traits and hypermasculinization for males and defeminization for females, consistent with the EMB theory.

## Introduction

Individuals with an autism spectrum disorder (ASD) are four times more likely to be male than female, and are also more likely than their typical counterparts to display a hypermasculinized (if male) or defeminized (if female) cognitive profile characterized by a stronger capacity for systemizing than empathizing [1]. These findings form the basis of Baron-Cohen [1] Extreme Male Brain (EMB) theory, which asserts that the behaviours seen in ASD are an exaggeration of typical sex-differences related to empathizing and systemizing [2].

Extending this account, Baron-Cohen and colleagues further argue that exposure to high levels of testosterone in utero contributes to the development of ASD [3, 4]. Support for the EMB theory comes from studies reporting associations between elevated prenatal testosterone levels recorded from either amniotic fluid or umbilical cord blood and key ASD symptoms including difficulties with eye contact, vocabulary development and social interaction [5–8]. Lower second-to-fourth digit ratios (purportedly related to exposure to high levels of prenatal testosterone; [9]) are also found amongst children with ASD [10]. Elevated prenatal steroidogenic activity has also been found in amniotic fluid samples for male foetuses where the boys were later diagnosed with ASD [11]. There is also additional evidence linking postnatal testosterone and ASD, including reports of elevated levels of androgen metabolites in postnatal serum samples for individuals with an ASD compared to controls [12], as well as increased incidence of females with ASD being diagnosed with testosterone-related disorders [13] such as polycystic ovary syndrome [14].

The proposed link between elevated levels of prenatal testosterone and autism spectrum traits has not been consistently supported in all studies. For example, there is some evidence that both males and females with gender identity disorder, rather than females only [15], are at increased risk of ASD [16]. From the EMB theory, one would predict that only females should encounter these issues. The findings reported by de Vries et al. [16] instead lend some support to Bejerot and colleagues’ [17] claim that ASD should be considered a ‘gender defiant’ disorder. This claim was based on their findings of androgyny in ASD, including elevated serum testosterone levels in ASD females and higher second-to-fourth digit (2D:4D) ratios (purportedly reflecting lower prenatal testosterone levels) in ASD males, relative to same-sex controls. Bejerot et al. [17] collected further evidence to bolster their claims through having the faces, voices, and body shapes of their ASD and control participants rated for gender coherence (using the scale: very gender-typical; gender-typical; average; weak gender coherence; very weak gender coherence). For the women in their study, Bejerot et al. [17] reported that gender coherence ratings for faces (but not voices or bodies) were lower in ASD cases compared to controls, and across the two groups, lower face gender coherence ratings correlated with higher scores on the Autism-spectrum Quotient (AQ; [18]). For the men, gender coherence ratings for voices and bodies (but not faces) were lower in ASD cases compared to controls, but only the body gender coherence ratings correlated negatively with AQ scores across groups. Thus the significant effects reported by Bejerot and colleagues [17] are consistent with linking ASD with androgyny.

The aim of the current study was to test the EMB and androgyny accounts of ASD by comparing non-clinical groups selected for high versus low AQ scores as to the rated masculinity (for male groups) or femininity (for female groups) of their faces and voices. We used masculinity and femininity ratings because of the well-evidenced links between higher testosterone levels and more masculinized or defeminized facial and vocal features [19–22]. These scales were preferred to the gender-coherence rating scale used by Bejerot et al. [17] which may be ambiguous as to whether ‘very gender-typical’ refers to an extreme level or instead an average (typical) level of masculinity for males/femininity for females. Further, the two points gender-typical and average appear similar in connotation. To control for extraneous sources of variance in perceived femininity/masculinity, the participants were all Caucasian and recruited from a narrow age range. Under the EMB account, individuals who score highly on the AQ should be rated as more masculine (if male) or less feminine (if female) than their low-AQ counterparts. If instead autism-spectrum traits are linked with androgyny, high-AQ males should be perceived as less masculine and high-AQ females as less feminine relative to their low-AQ comparison groups.

## Materials and Methods

### Participants

#### Face stimuli

A cohort of 76 Caucasian undergraduate students accepted invitations to participate in a session in which they had their faces photographed. These students had been identified as High (≥ 20) or Low (≤ 12) AQ scorers in other studies conducted by our research group. The cut-offs used correspond to approximately the 25^th^ and 75^th^ percentiles of the combined-sex AQ distribution in Western Australian university samples, using the original 0/1 method of scoring [23].

#### Voice stimuli

A second cohort of 47 Caucasian High- and Low-AQ students, fluent in English, was recruited in a similar manner to provide voice samples. Descriptive statistics for the two cohorts are presented in Table 1.

**Table 1:** Descriptive statistics for the pairs of High and Low AQ groups for each sex. Note. z-scores were calculated with reference to the distributions of AQ scores for all males (N = 440; overall mean AQ = 16.39) and females (N = 931; overall mean AQ = 15.59) screened in this study.

	Males		Females	
	High AQ	Low AQ	High AQ	Low AQ
Faces				
N	17	17	21	21
AQ				
Mean (SD)	26.8 (4.8)	9.3 (1.6)	26.4 (3.1)	8.0 (2.2)
z-score	1.68	-1.15	1.89	-1.33
Range	20 to 38	5 to 11	21 to 35	4 to 12
Age (years)				
Mean (SD)	17.8 (.10)	18.0 (1.0)	17.7 (.72)	17.7 (.73)
Voices				
N	11	12	12	12
AQ				
Mean (SD)	24.7 (3.0)	9.8 (1.2)	28.0 (3.0)	7.2 (1.7)
z-score	1.34	-1.07	2.17	-1.47
Range	20 to 33	8 to 12	24 to 35	4 to 9
Age (years)				
Mean (SD)	18.6 (1.4)	18.2 (1.2)	17.83 (.8)	17.3 (.5)

#### Raters

Thirty Caucasian undergraduate students (15 of each sex; mean age = 18.9 years; SD = 2.9 years) were additionally recruited to rate the masculinity and femininity of the faces and voices.

### Ethics

The recruitment and testing of all participants was conducted in accordance with the ethical approval obtained for this study from the Human Research Ethics Committee at the University of Western Australia. In accordance with the ethics approval obtained, all participants signed a written consent form prior to taking part in the study.

### Procedure

#### Questionnaire

The 50-item Autism-spectrum Quotient [18] was used to assess participants’ levels of autistic-like traits (score range 0–50).

#### Face photography

Each participant in the first cohort was photographed front-on while seated 3m from a Canon digital single-lens reflex camera placed on a tripod. Participants were instructed to wear no make-up or jewellery, remove glasses, pin any loose hair away from the face, and hold a neutral facial expression. All males were clean-shaven. Adobe Photoshop was used to rotate each face so the pupil centres were horizontal and separated by 80 pixels. An ellipse was drawn over the face so that everything outside the hairline, ears, and just below the chin was obscured with a grey frame. Photographs were presented as 320 x 420 pixel black-and-white images.

#### Voice recording

In a soundproof room, each participant in the second cohort read the Rainbow Passage [24] using a conversational tone. Sound Forge 4.5 was used to extract the second sentence and normalize its intensity in preparation for the masculinity/ femininity rating task.

#### Masculinity/femininity ratings

The 30 students rated the faces and voices for their femininity (female stimuli) or masculinity (male stimuli) in four blocks (female faces, female voices, male faces, male voices), with block order counterbalanced across raters. Within each block, the face/voice stimuli were presented in random order. Following the presentation of each face centrally on the computer screen (for 5s) or each voice through enclosed headphones, a rating scale appeared on the screen. The scale ranged from 1 to 10, with the extreme points labelled ‘Not at all masculine’ and ‘Extremely masculine’ for male stimuli, and ‘Not at all feminine’ and ‘Extremely feminine’ for female stimuli.

## Results

**Fig 1:**
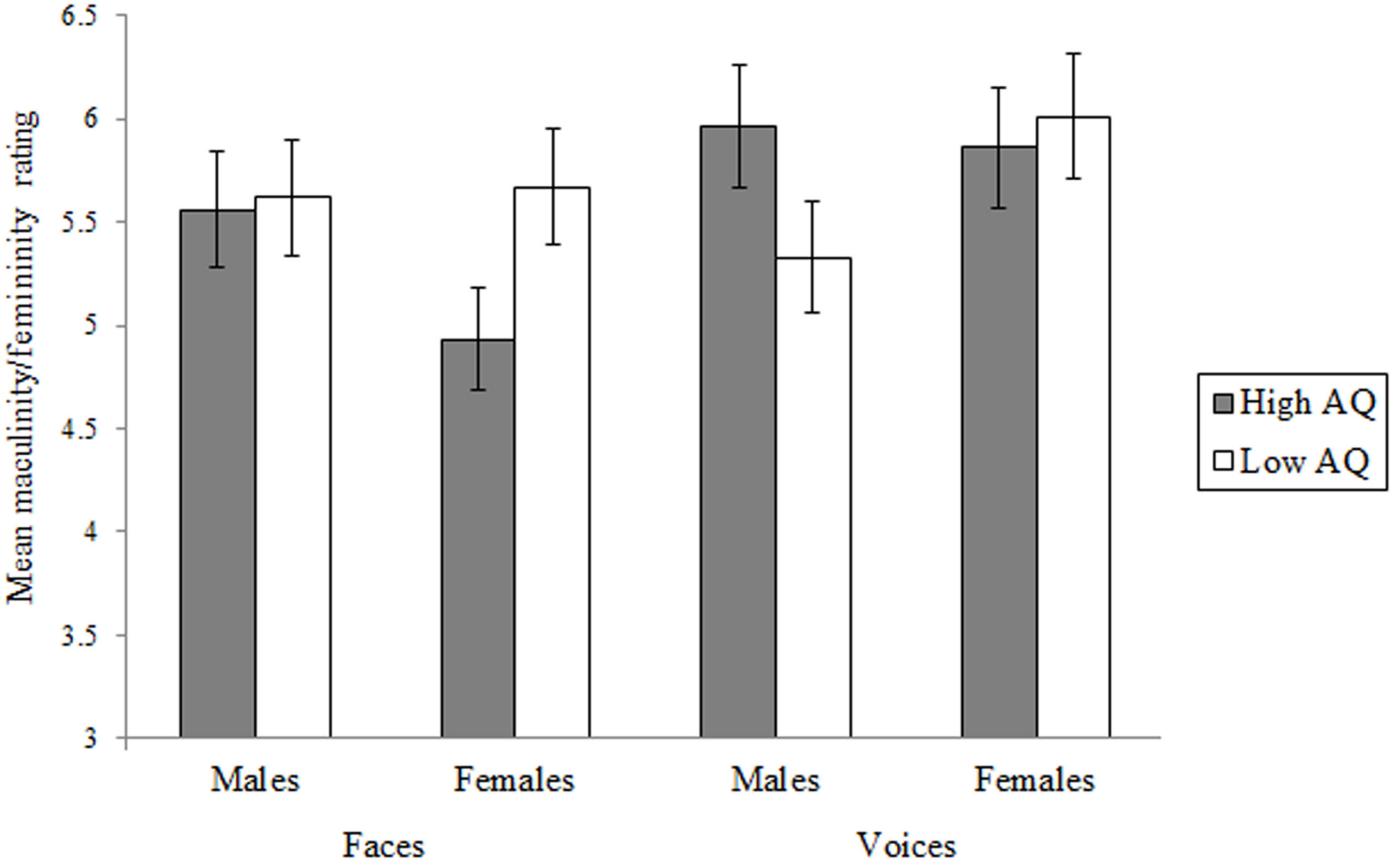
Mean masculinity and femininity ratings (with 95% CIs) for the High and Low AQ groups for face and voice.

Examining the distributions of ratings for each of the faces and voices revealed one outlying rating (deviating from the group mean by more than 3.29 SDs) which was adjusted as recommended by Tabachnick and Fidell [25]. For each rater, mean ratings were calculated for the high and low AQ groups in each of the four stimulus sets (i.e., for the male and female faces and voices). Single-factor repeated-measures ANOVAs were then conducted on these data to examine whether High-AQ male participants were rated as having more masculinized faces and/or voices than their Low-AQ counterparts. The ratings obtained for the females were analysed in the same way to determine whether the High-AQ females were rated as more feminized in their faces and/or voices than the Low-AQ females (see Fig 1 for means). These analyses revealed a significant effect of AQ group for the female faces, with High-AQ faces rated as less feminine than Low-AQ faces, F(1, 29) = 50.18, p < .001, η_p_^2^ = .63 (range of ratings for high-AQ females = 3.24 to 6.71, and low-AQ = 3.62 to 7.38). The effect of AQ group for masculinity ratings of the male faces was not significant, F(1, 29) = .49, p = .49, η_p_^2^ = .02 (range of ratings for high-AQ males = 4.29 to 6.71, and low-AQ = 4.35 to 6.35). For voices, High-AQ males were rated as more masculine than Low-AQ males, F(1, 29) = 36.20, p < .001, η_p_^2^ = .55 (range of ratings for high-AQ males = 4.18 to 7.64, and low-AQ = 4.17 to 6.92), while the femininity ratings for High- and Low-AQ female voices did not differ significantly, F(1, 29) = 1.74, p = .20, η_p_^2^ = .06 (range of ratings for high-AQ females = 4.75 to 8.17, and low-AQ = 4.42 to 8.58).

The ratings of faces had high reliabilities with Cronbach’s α = .97 for male faces and .96 for female faces. Voice ratings also had high reliabilities with Cronbach’s α = .98 for male voices and .95 for female voices. Pearson correlation analysis conducted for each AQ group for each sex showed no significant relationships between AQ scores and ratings of faces and voices.

## Discussion

The findings of less feminine faces for higher AQ females and more masculinized voices for higher AQ males are consistent with the EMB theory of Baron-Cohen et al. [1]. While the lower femininity ratings for the faces of High-AQ women could also be consistent with the claim of Bejerot and colleagues [17] that autistic traits are associated with androgynous physical characteristics, the outcome of higher masculinity ratings for the voices of High-AQ men is contrary to their account.

In weighing up the level of support the current findings provide for masculinization accounts of ASD, it is important to consider why, if elevated testosterone levels are associated with autistic features, effects of AQ group were not found consistently for face and voice. To speculate, one possibility is that testosterone affects these features to differing degrees in males and females, and the more subtle differences were not detected by raters. This explanation would be in keeping with Lippa’s [26] findings which suggest that raters tend to use physical appearance cues more when making femininity judgments, and vocal cues more when making masculinity judgements. These findings offer a neat explanation for the results obtained in the current study, with raters being more attuned to differences in the physical appearance of High- and Low-AQ females, and differences in the vocal characteristics of High- and Low-AQ males.

The different patterns of findings for the ratings of the present study and those reported by Bejerot et al. [17] could reflect any of several differences in method. While the use of nonclinical versus clinical samples may be important, there is now good evidence of continuity in behaviour and aetiology across groups with high but subclinical levels of mild autistic traits and groups with an ASD diagnosis [18, 27]. The use of different rating scales may also have contributed to the divergent results. The masculinity/femininity ratings of the current study are arguably less ambiguous and relate more directly to the EMB and androgyny accounts than do the gender-coherence ratings of Bejerot and colleagues [17]. Finally, whereas the High- and Low-AQ groups of the current study were drawn from a common pool of volunteers and were well-matched on age, education and ethnicity, we did not measure other variables of potential relevance such as height, weight and BMI. On the other hand, the ASD and control groups studied by Bejerot et al. [17] were drawn (necessarily) from different pools of volunteers, and differed significantly on level of education, height, weight, and BMI, variables that may affect ratings of faces, voices and bodies.

In a recent study, Scott, Jones, Kramer, and Ward [28] reported an association between perceived masculinity and AQ scores for male faces but not for female faces. These results are somewhat consistent with the EMB theory, as are the findings in the current study. However, in the current study, significant results were reported for female faces only instead of male faces only, as in Scott et al. [28]. While the direction of relationship is similar for the two studies, the difference in findings could be due to methodological differences. Firstly, Scott et al. [28] used composite images derived from face photographs of high- and low-AQ scorers whereas in the current study, a more direct approach in using actual face photographs of high- and low-AQ scorers was employed. Secondly, masculinity ratings were used for both male and female faces in Scott et al. [28] whereas masculinity ratings were used only for male faces, with femininity ratings used for female faces, in the current study.

At the present time the broader literature provides more evidence for the EMB account of ASD than the androgyny account. For example, regardless of their sex, individuals at the upper end of the autism spectrum (both in the clinical and non-clinical ranges) tend to outperform controls on tasks typically favouring males, but tend to underperform controls on tasks typically favouring females (e.g., [29, 30]; for reviews see [1, 31]. Also, contrary to the results of Bejerot and colleagues [17], recent meta-analyses report evidence of significantly lower 2D:4D ratios (consistent with higher levels of prenatal testosterone exposure) in both male and female ASD samples relative to controls [32, 33]. There are also several studies reporting elevated postnatal levels of androgens in either serum or saliva for ASD cases relative to controls, including a study in which differences were more pronounced in males [12, 34]. This said, Bejerot et al. do review some evidence inconsistent with the EMB account of autism, including the higher incidence of gender-identity disorder in ASD males compared to ASD females [16].

To our knowledge, this is the first study to explore degree of perceived masculinity and femininity of the faces and voices of typical Caucasian adults selected for high and low levels of autistic-like traits. However, there are some caveats to be borne in mind while interpreting the reported data. Firstly, although extraneous variables such as ethnicity of high- and low-AQ scorers and raters were controlled (i.e., limited to Caucasian participants only), there was a lack of information about the sexual orientation of the AQ scorers and face/voice raters which might introduce variability to the ratings. Secondly, it is possible for participants with faces that are deemed as “unusual” to be rated as less feminine or masculine. Nonetheless, there is good evidence that ratings of masculinity and femininity are strongly influenced by sexually dimorphic facial features [35, 36]. Thirdly, the sample sizes in the current are modest, hence a more highly-powered follow-up study (larger sample size and wider separation of AQ groups) is warranted. Lastly, the current study did not examine ratings of the faces and voices of individuals clinically diagnosed with ASD. The current findings warrant future research that examines if the pattern of results for the current study extends to the clinical population.

In summary, our key findings—that higher levels of autistic traits are associated with more masculinized voices in males and defeminized facial features in females—provide some support for the EMB account of ASD [1, 37]. The results reported in the current paper are preliminary and further investigation of relationships between autistic traits and features of the face and voice would benefit from supplementing masculinity and femininity ratings with objective measures such as morphological (e.g., using 3D face photographs) and vocal (e.g., pitch) analyses. It would also be of benefit to assess androgen levels at several points in development given that pre-pubertal and post-pubertal testosterone levels appear to differentially influence aspects of facial shape that contribute to a more feminine or more masculine facial appearance [38].
